# Activation of the beta interferon promoter by paramyxoviruses in the absence of virus protein synthesis

**DOI:** 10.1099/vir.0.037531-0

**Published:** 2012-02

**Authors:** M. J. Killip, D. F. Young, B. L. Precious, S. Goodbourn, R. E. Randall

**Affiliations:** 1 School of Biology, Centre for Biomolecular Sciences, BMS Building, North Haugh, University of St Andrews, St Andrews, Fife KY16 9ST, UK; 2 Division of Basic Medical Sciences, St George’s, University of London, London SW17 0RE, UK

## Abstract

Conflicting reports exist regarding the requirement for virus replication in interferon (IFN) induction by paramyxoviruses. Our previous work has demonstrated that pathogen-associated molecular patterns capable of activating the IFN-induction cascade are not normally generated during virus replication, but are associated instead with the presence of defective interfering (DI) viruses. We demonstrate here that DIs of paramyxoviruses, including parainfluenza virus 5, mumps virus and Sendai virus, can activate the IFN-induction cascade and the IFN-β promoter in the absence of virus protein synthesis. As virus protein synthesis is an absolute requirement for paramyxovirus genome replication, our results indicate that these DI viruses do not require replication to activate the IFN-induction cascade.

## Introduction

Viruses activate the interferon (IFN) response in infected cells through the generation or exposure of molecular structures, termed pathogen-associated molecular patterns (PAMPs), which are absent in uninfected cells. During negative-sense RNA virus infections, these PAMPs are detected by the cytoplasmic RNA helicases RIG-I and MDA-5, which activate a downstream signalling cascade culminating in the activation of ATF2/c-Jun, NF-κB and IRF3 transcription factors, and subsequent transcription of the IFN-β gene (reviewed by Kumar *et al.*, 2011; Onomoto *et al.*, 2010). Secreted IFN establishes an ‘antiviral state’ in both the infected cell and neighbouring uninfected cells by upregulating a large number of antiviral IFN-stimulated genes (ISGs) (reviewed by Randall & Goodbourn, 2008). The IFN response is very effective at limiting virus replication and spread; to replicate efficiently, therefore, viruses encode antagonists of the IFN response in order to limit IFN induction or the ability of IFN to exert its antiviral effects.

Paramyxoviruses are usually poor activators of the IFN response, but virus preparations that are rich in defective interfering (DI) viruses are potent inducers of IFN-β (Chen *et al.*, 2010; Johnston, 1981; Killip *et al.*, 2011; Poole *et al.*, 2002; Shingai *et al.*, 2007; Strahle *et al.*, 2006). DI viruses are unable to complete a full replication cycle due to genome deletions and consequently require co-infecting non-defective (ND) viruses to supplement the missing viral factors needed to replicate their genomes. DIs can also interfere with the replication of ND viruses through competition for viral or host factors essential for replication or because of their replicative advantage due to the smaller size of their genome (reviewed by Marriott & Dimmock, 2010; Re, 1991).

We have recently developed a GFP reporter cell line that faithfully reports activation of the IFN-induction cascade and the IFN-β promoter in individual cells (Chen *et al.*, 2010). Using this cell line, we have demonstrated that heterocellular IFN-β promoter activation occurs during infection with parainfluenza virus 5 (PIV5), even following infection with a recombinant PIV5 that lacks a functional IFN antagonist (Killip *et al.*, 2011). This suggests strongly that PIV5 does not normally generate or expose PAMPs capable of activating the IFN-induction cascade during its replication cycle. Furthermore, we demonstrated that IFN-β promoter activation in individual infected cells infected with PIV5 or mumps virus (MuV) correlated with the presence of DI viruses in virus preparations, indicating that DIs are predominantly responsible for inducing IFN during infections with these viruses (Chen *et al.*, 2010; Killip *et al.*, 2011). Here we demonstrate that neither a co-infecting ND virus nor virus protein synthesis (and therefore genome replication) is required for the activation of the IFN-induction cascade by paramyxovirus DIs.

## Results

### IFN-β promoter activation by PIV5 DI viruses is not correlated with the level of virus replication

**Fig. 1.**
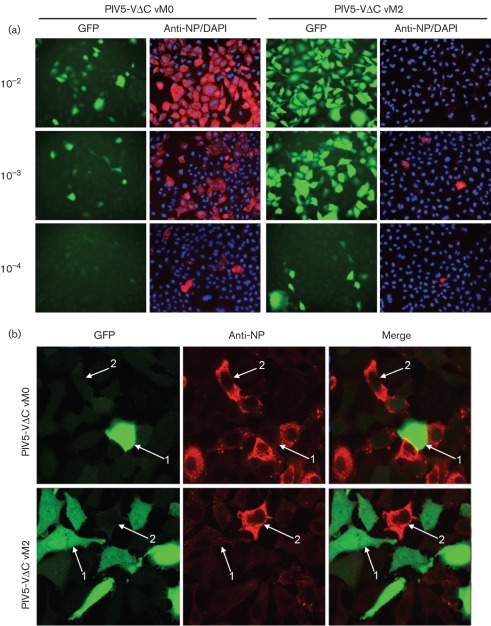
PIV5-VΔC DIs activate the IFN-β promoter in the absence of a co-infecting ND virus. (a) A549/pr(IFN-β).GFP cells were infected with 10^−2^, 10^−3^ or 10^−4^ dilutions of PIV5-VΔC vM0 or vM2. Eighteen hours later, monolayers were fixed and immunostained for virus NP expression. GFP and NP (red) were visualized by fluorescence microscopy. Nuclear material was stained with DAPI (blue). (b) A549/pr(IFN-β).GFP cells were infected with 0.1 p.f.u. PIV5-VΔC vM2 or vM0 per cell. Cells were fixed at 18 h post-infection and immunostained for virus NP expression. GFP and NP (red) were visualized by confocal microscopy. Selected cells within the panels are highlighted by white arrows; cells that are strongly positive for GFP but very weakly NP-positive are labelled 1; 2 denotes cells strongly positive for virus NP, but negative for GFP.

Our previous work has demonstrated that a recombinant PIV5 virus, PIV5-VΔC, that lacks IFN-antagonist activity due to a C-terminal deletion in its V protein, does not activate the IFN-β promoter in the majority of infected cells unless it has been prepared by sequential high-multiplicity passages, a process that causes the accumulation of DI viruses (Killip *et al.*, 2011). Thus, a DI-rich preparation of PIV5-VΔC [termed vM2 after the von Magnus effect (von Magnus, 1951) and the two sequential high-multiplicity passages required to generate the stock] was very efficient at inducing IFN compared with our original (vM0) PIV5-VΔC preparation (Killip *et al.*, 2011). The PIV5-VΔC vM2 stock was also efficient at inhibiting the replication of ND PIV5, consistent with the interfering nature of DI viruses. Subsequent next-generation sequencing of RNA isolated from CsCl-purified nucleocapsids has revealed that the number of trailer copyback DIs (Killip *et al.*, 2011) was 20–40 times greater than that of ND genomes in the PIV5-VΔC vM2 preparation, whilst ND genomes predominated over DI genomes in the vM0 stock (unpublished data). Thus, the observation that the vM2 preparation was significantly better at inducing IFN than the vM0 preparation suggested a lack of correlation between IFN induction and virus replication. To examine the relationship between these two variables more closely, A549/pr(IFN-β).GFP reporter cells were infected with increasing dilutions of PIV5-VΔC vM0 or PIV5-VΔC vM2. The levels of GFP and virus NP were subsequently determined by immunofluorescence (Fig. 1a). At each dilution of PIV5-VΔC vM0 or PIV5-VΔC vM2, cells that were strongly positive for virus NP (indicating normal virus replication) were usually negative for GFP, whereas those that were strongly positive for GFP were generally only very weakly NP-positive. Additionally, as will be discussed further below, GFP-positive cells could clearly be observed even at high dilutions (10^−4^) of virus, where very few cells would have been infected with an ND (i.e. plaque-forming) virus. Confocal photomicrographs in Fig. 1(b) show very clearly, at a higher magnification, the lack of correlation between GFP and virus protein expression. In cells infected with PIV5-VΔC vM2, whilst the majority of cells were positive for GFP expression, the same cells were generally only weakly NP-positive. In contrast, following infection with PIV5-VΔC vM0, the majority of A549/pr(IFN-β).GFP cells were strongly positive for NP, but were negative for GFP expression. Thus, for both DI-rich (PIV5-VΔC vM2) and DI-poor (PIV5-VΔC vM0) infections, very little (if any) virus protein synthesis and thus replication was occurring in those cells in which the IFN-β promoter had been activated.

### IFN-β promoter activation by PIV5 DIs does not require co-infection with ND virus

GFP-positive cells could still clearly be observed following infection with high dilutions (10^−4^) of PIV5-VΔC vM2 virus (Fig. 1a). This dilution of virus corresponded to approximately 0.01 p.f.u. per cell, an m.o.i. at which very few cells are infected with an ND virus particle. The vast majority of cells were therefore unlikely to have been co-infected with both a DI and an ND virus, suggesting that PIV5-VΔC DIs do not require a co-infecting ND virus particle to activate the IFN-β promoter. To investigate this further, we determined the percentage of GFP-positive cells following infection with increasing dilutions of PIV5-VΔC vM2 by FACS and compared this with the percentage of cells that would be expected to be infected with ND virus at each dilution as predicted using the Poisson distribution (Table 1; Supplementary Fig. S1, available in JGV Online). At all dilutions of PIV5-VΔC vM2 examined, the percentage of GFP-positive cells was higher than the predicted percentage of ND-infected cells. For example, at a dilution of 10^−3^, only 9.5 % of cells were predicted to be productively infected, whereas 35.7 % of cells were positive for GFP by FACS, indicating clearly that PIV5-VΔC DIs can activate the IFN-β promoter in the absence of a co-infecting ND virus.

**Table 1.** Activation of the IFN-β promoter by different multiplicities of PIV5-VΔC vM2 Duplicate dilutions of PIV5-VΔC vM2 were titrated on Vero cells or used to infect A549/pr(IFN-β).GFP cells in order to determine GFP expression by FACS 16 h later. The percentage of cells expected to be infected with an ND virus at each dilution was calculated using the Poisson distribution.

Dilution of PIV5-VΔC vM2	m.o.i. (p.f.u. per cell)	*P*(0)* (%)	*P*(≥1)† (%)	GFP-positive cells (%)
10^−2^	1	36.8	63.2	75.1
10^−3^	0.1	90.5	9.5	35.7
10^−4^	0.01	99.0	1.0	7.9

* The probability of a cell remaining uninfected by a plaque-forming particle, *P*(0), was defined as *P*(0) = e^−^*^m^* (where *m* = m.o.i.) and expressed as a percentage. Further explanation of the derivation of this formula is given in Methods.

† The probability of a cell being infected by ≥1 p.f.u. was defined as *P*(≥1) = 1−*P*(0) and expressed as a percentage.

### PIV5 DIs activate IRF3 and the IFN-β promoter in the absence of virus protein synthesis

**Fig. 2.**
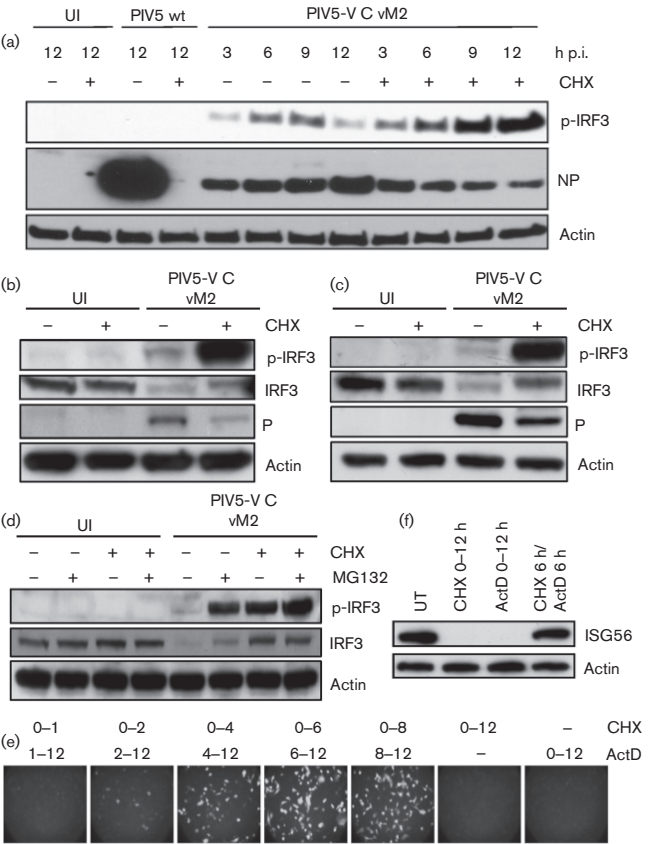
PIV5-VΔC DIs can activate IRF3 and the IFN-β promoter in the absence of virus protein synthesis. (a) A549 cells were mock-infected or infected with 10 p.f.u. PIV5 wt vM0 or PIV5-VΔC vM2 per cell in the presence or absence of cycloheximide (CHX). At various times p.i., the cells were harvested and the presence of phosphorylated IRF3 (p-IRF3), viral NP and actin was detected by immunoblot analysis. Uninfected (UI) cells were included as a negative control. Note: for PIV5-VΔC vM2 infections, the amount of NP corresponds to input virions only, as PIV5-VΔC DIs inhibit ND virus replication significantly in infected cells. As a result, although CHX markedly reduces PIV5 wt NP synthesis, it does not significantly affect NP expression in PIV5-VΔC vM2-infected cells. (b, c) MRC-5 (b) or HSF (c) cells were mock-infected or infected with 10 p.f.u. PIV5-VΔC vM2 per cell in the presence or absence of CHX. Sixteen hours p.i., cells were harvested and lysates were immunoblotted for p-IRF3, total IRF3, viral P protein and actin. (d) A549 cells were infected with 10 p.f.u. PIV5-VΔC vM2 per cell in the presence of CHX and/or the proteasome inhibitor MG132. Sixteen hours p.i., cells were harvested and lysates were immunoblotted for p-IRF3, total IRF3 and actin. (e) A549/pr(IFN-β).GFP cells were infected with 10 p.f.u. PIV5-VΔC vM2 per cell and cultured in the presence of CHX for the times indicated, after which the CHX block was removed and any further transcription was prevented by culturing the cells in medium containing actinomycin D (ActD). At 12 h p.i., GFP-positive cells were visualized by fluorescence microscopy. (f) A549 cells were infected with 10 p.f.u. PIV5-VΔC vM2 per cell in the presence of CHX or ActD for 12 h, or 6 h CHX followed by 6 h ActD treatment. Monolayers were harvested and ISG56 and actin were detected by immunoblot analysis.

Virus protein synthesis is an absolute necessity for both DI and ND paramyxovirus genome replication due to the requirement for concurrent nucleocapsid assembly (Horikami *et al.*, 1992; Vidal & Kolakofsky, 1989). The observation that the PIV5-VΔC-infected cells that were GFP-positive were usually negative for virus protein expression suggested that virus protein synthesis and replication may not always be required to induce IFN. We therefore sought to determine whether DI-rich PIV5-VΔC virus preparations could activate the IFN-induction cascade following treatment with protein synthesis inhibitors. To this end, A549 cells were infected with PIV5-VΔC vM2 in the presence or absence of cycloheximide (CHX), and active, phosphorylated IRF3 (p-IRF3) was detected by immunoblot analysis of harvested lysates. Following treatment with 50 µg CHX ml^−1^, a concentration that inhibits both cellular and viral protein synthesis (Fig. 2a), p-IRF3 could be detected within 3 h of PIV5-VΔC vM2 infection, demonstrating that PIV5-VΔC vM2 infection activates IRF3 and therefore the IFN-induction cascade in the absence of either cellular or viral protein synthesis. The ability of PIV5-VΔC vM2 to activate IRF3 in the presence of CHX was not an effect that was limited to A549 cells, as we observed similar results in other cell types, including untransformed human lung cells (MRC-5; Fig. 2b) and untransformed human skin fibroblasts (HSF; Fig. 2c). An additional striking observation from these experiments was that p-IRF3 levels were considerably higher in cells infected in the presence of CHX than in untreated cells. Further experiments revealed that treatment with the proteasome inhibitor MG132 could restore p-IRF3 levels to those seen following CHX treatment. Furthermore, a decrease in total IRF3 levels could be observed following infection with PIV5-VΔC vM2, which could be prevented by treatment with either CHX or the proteasome inhibitor MG132 (Fig. 2d). These data are consistent with the proteasome-mediated degradation of activated IRF3 during virus infection (in order to prevent continuous activation of the IFN-β promoter) by a mechanism that requires ongoing protein synthesis (Lin *et al.*, 1998; Ye & Maniatis, 2011).

We next determined whether the promoters of IFN-β and other IRF3-responsive genes could be activated in the absence of virus protein synthesis. A549/pr(IFN-β).GFP cells were infected with PIV5-VΔC vM2 in the presence of CHX and, at various times post-infection (p.i.), the CHX block was reversed in the presence of actinomycin D (to prevent any further transcription of cellular genes). The cells were cultured until 12 h p.i. and subsequently examined for the presence of GFP-positive cells; under these conditions, any GFP synthesized must have been made from mRNAs that accumulated in the presence of CHX, and therefore in the absence of virus protein synthesis. Fig. 2(e) shows that GFP-positive cells could readily be detected under these conditions, confirming that the IFN-β promoter must have been activated prior to the onset of virus protein synthesis. Furthermore, our results were not restricted to our GFP reporter gene, as we could also detect expression of the IRF3-upregulated ISG56 protein under the CHX/actinomycin D reversal conditions (Fig. 2f). These results demonstrate that PIV5-VΔC DIs can activate the IFN-induction cascade and the IFN-β promoter in the absence of virus protein synthesis, and therefore virus genome replication.

### Virus protein synthesis is not required for IRF3 activation by DI-rich stocks of other paramyxoviruses

**Fig. 3.**
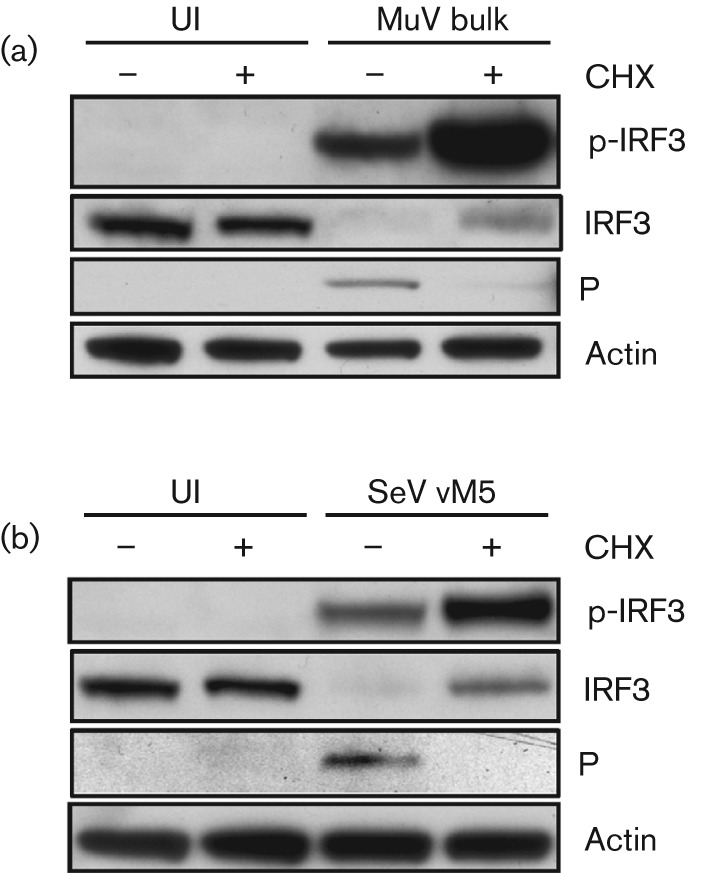
Virus protein synthesis is not required to activate the IFN-induction cascade by DI-rich preparations of other paramyxoviruses. (a) A549 cells were uninfected (UI) or infected with a DI-rich preparation of MuV (MuV bulk) in the presence or absence of CHX. Twelve hours later, monolayers were harvested, and p-IRF3, viral P and actin were detected by immunoblot analysis. (b) As for (a), but with SeV vM5 preparation.

DI-rich stocks of other paramyxoviruses, including MuV and Sendai virus (SeV), have previously been shown to be good inducers of IFN (Chen *et al.*, 2010; Johnston, 1981; Strahle *et al.*, 2006). To determine whether virus protein synthesis was also dispensable for IFN induction by these viruses, we repeated our CHX experiments with DI-rich stocks of MuV (termed MuV bulk) and SeV (vM5). As we observed above for PIV5-VΔC, both MuV bulk and SeV vM5 activated IRF3 strongly in the presence of CHX (Fig. 3a, b), indicating that DIs of other paramyxoviruses can activate the IFN-induction cascade without virus protein synthesis and genome replication.

### Virus protein synthesis is required for optimal activation of IRF3 by virus preparations that have not been enriched for DIs

**Fig. 4.**
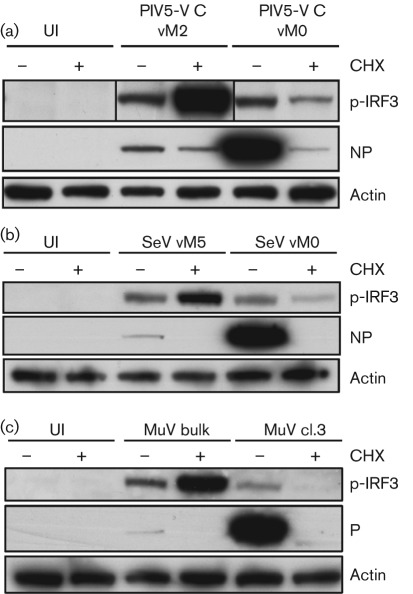
Replication is required for maximal activation of the IFN-induction cascade by paramyxovirus preparations that have not been deliberately enriched for DIs. A549 cells were uninfected (UI) or infected with 10 p.f.u. per cell of PIV5-VΔC vM2 or vM0 (a), SeV vM5 or vM0 (b), or MuV bulk or cl.3 (c) in the presence or absence of CHX. Sixteen hours later, monolayers were harvested, and p-IRF3, viral protein (either P or NP, as indicated) and actin were detected by immunoblot analysis. Note: the p-IRF3 panel for PIV5-VΔC has been spliced to modify the order of the lanes.

We have shown above that DI-rich paramyxovirus preparations strongly activate the IFN-induction cascade and the IFN-β promoter in the presence of protein synthesis inhibitors. We investigated next whether preparations of paramyxoviruses that were generated by low-multiplicity passage, and therefore were not deliberately enriched for DI viruses (i.e. our working stocks of virus), could activate the IFN-induction cascade in the absence of virus protein synthesis. IRF3 activation could be detected in untreated cells infected with our vM0 preparations of PIV5-VΔC and SeV, but, in contrast to infections with DI-rich preparations of these viruses, treatment with CHX had an inhibitory effect on IRF3 activation in infected cells (Fig. 4a, b). For these virus preparations, therefore, inhibition of virus protein synthesis limited activation of the IFN-induction cascade. This inhibition was not complete, however, and small amounts of p-IRF3 were detectable even in the presence of CHX. This low-level activation of the IFN-induction cascade by these virus stocks in the absence of protein synthesis suggests that DIs are present even in virus preparations that have been generated so as to minimize DI generation. Our working stock of MuV (termed MuV cl.3) was generated by plaque purification of our DI-rich MuV preparation (MuV bulk) [for further characterization, see Chen *et al.* (2010)] and could therefore be considered ‘DI-poor’; consistent with this, IRF3 phosphorylation was not detectable in MuV cl. 3-infected cells in the presence of CHX (Fig. 4c). Replication was required for IRF3 activation by this virus preparation, as p-IRF3 was detectable in untreated but not CHX-treated cells.

We next examined transcription of the IFN-β gene at various times p.i. with SeV in the presence or absence of CHX. At 2 h p.i., levels of IFN-β transcript were similar in untreated or CHX-treated cells (Supplementary Fig. S2, available in JGV Online). At later times, however, transcription of IFN-β increased steadily in untreated cells between 4 and 8 h p.i., whereas levels of IFN-β transcript remained stable in CHX-treated cells. As a result, IFN-β transcription was considerably higher in untreated than in CHX-treated cells by 8 h p.i. These results are consistent with DIs already being present in this SeV preparation, inducing IFN in the absence of virus protein synthesis and replication. However, if virus replication is allowed to proceed, activation of the IFN-induction cascade increases as more PAMPs are generated, presumably due to the amplification of existing DIs and/or the *de novo* generation of DIs during genome replication.

### Virus binding is insufficient to activate the IFN-β promoter

**Fig. 5.**
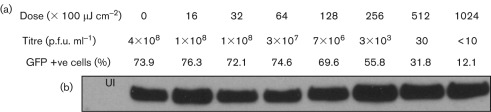
Effect of UV treatment on PIV5-VΔC infectivity, virus binding and IFN-β promoter activation. (a) Two hundred and twenty microlitres of a 10^−1^ dilution of PIV5-VΔC vM2 was treated with doses of UV radiation as indicated. Following treatment, infectious virus was titrated by plaque assay on Vero cells, and FACS analysis was used to measure the percentage of GFP-positive cells following infection of A549/pr(IFN-β).GFP cells with the UV-treated virus. The effect of UV radiation on the ability of the treated virus to bind to infected cells is shown. (b) A549/pr(IFN-β).GFP cells were uninfected (UI) or infected with 100 p.f.u. per cell of PIV5-VΔC vM2 that had been treated with UV light as detailed above in the presence of 50 µg CHX ml^−1^. At 2 h p.i., the cells were harvested and viral NP was detected by immunoblot analysis.

The data presented above demonstrate clearly that the IFN-β promoter can be activated by paramyxovirus DIs in the absence of virus protein synthesis. It is also clear that virus binding is a prerequisite for IFN induction, as IFN induction by PIV5-VΔC vM2 is prevented completely by treatment with neutralizing antibody to virus haemagglutinin–neuraminidase (Supplementary Fig. S3, available in JGV Online). Furthermore, this result also rules out that the IFN induction seen with this virus is caused by free RNA present in the virus inoculum. It has previously been reported that binding and entry of some enveloped viruses, e.g. herpesviruses, can activate an IRF-dependent antiviral response that is triggered by increasing amounts of input virus (Mossman *et al.*, 2001; Preston *et al.*, 2001). This was unlikely to be the case for PIV5, as we observed activation of the IFN-induction cascade even at very low multiplicity (Fig. 1a; Table 1). Nevertheless, we tested whether virus binding alone was sufficient to trigger the IFN-induction cascade using a UV-inactivation approach; UV exposure impairs virus replication by inducing uracil dimers in regions of the RNA genome that prevent the progress of viral RNA-dependent RNA polymerase (Ball & White, 1976). PIV5-VΔC vM2 was treated with a range of doses of UV irradiation (from 0 to 102 400 µJ cm^−2^), and the ability of the UV-treated virus to induce GFP expression in A549/pr(IFN-β).GFP reporter cells was correlated with virus infectivity and the ability of UV-treated virus to bind to cells. Although high doses of UV irradiation can affect protein function and hence virus binding (Miller & Plagemann, 1974), it was clear that PIV5-VΔC vM2 virus binding to cells was not reduced over the range of UV treatment used (Fig. 5b). However, over the same treatment range, the number of GFP-positive cells reduced from approximately 80 % to approximately 12 % (Fig. 5a). It is also of note that virus infectivity was much more sensitive to UV inactivation than the ability to activate the IFN-β promoter, providing further evidence that ND virus replication is not required for activation of the IFN-induction cascade by PIV5-VΔC DIs. For example, 25 600 µJ UV irradiation cm^−2^ reduced virus infectivity by greater than five orders of magnitude, but only reduced the number of GFP-positive cells from approximately 80 to 55 %. Taken together, these results demonstrate clearly that virus replication is not a prerequisite for the activation of IRF3 or the IFN-β promoter, and that the binding of PIV5 DI viruses to cells is also not directly responsible for activation of the IFN response. However, given the sensitivity of GFP induction to higher doses of UV treatment, the integrity of the virus genome is still required for IFN-β promoter activation. Therefore, it seems likely that viral RNA products made from incoming DI virus genomes, in the absence of virus protein synthesis or the DI genomes themselves, are responsible for activating the IFN-induction cascade.

## Discussion

Through single-cell studies of IFN-β promoter activation, we have demonstrated previously that PAMPs capable of activating the IFN-induction cascade are not normally generated during PIV5 replication, even when this virus lacks a functional IFN antagonist. We further demonstrated that IFN induction by virus stocks correlated with the presence of DI viruses, and therefore concluded that IFN induction was predominantly associated with the presence of DI viruses (Chen *et al.*, 2010; Killip *et al.*, 2011). Here, we have addressed the question of the requirement for paramyxovirus replication in IFN induction. We demonstrate that DI-rich preparations of several paramyxoviruses, including PIV5, MuV and SeV, induce IFN in the absence of virus protein synthesis and hence in the absence of genome replication. Conflicting reports exist in the literature regarding the requirement of virus replication for IFN induction by paramyxoviruses. Our results are consistent with studies of Newcastle disease virus (NDV), demonstrating that this virus does not require replication to induce an antiviral response (Collins *et al.*, 2004; Dianzani *et al.*, 1970). In addition, studies using measles virus demonstrated that IRF3 activation and the transcription of IFN-β mRNA occurred prior to the onset of virus protein synthesis (Shingai *et al.*, 2007), and that the kinetics of IFN-β transcription did not correlate with the rate of viral genome replication (Plumet *et al.*, 2007). However, although we have demonstrated clearly that DI-rich SeV preparations can activate the IFN-induction cascade in the absence of virus replication, a previous study of IFN induction by SeV DIs concluded that purified SeV DIs were unable to induce IFN unless infected simultaneously with ND virus (Johnston, 1981), whilst another showed that IFN induction required both genome replication and a co-infecting ND virus (Strahle *et al.*, 2006). As DIs generated during normal virus replication are likely to be a heterogeneous population, it would be very interesting to determine whether certain DI species within a population, but not others, require replication of their genome or a co-infecting ND virus to induce IFN.

A recent study has suggested that the RIG-I ligands in SeV-infected cells are progeny viral genomes (Rehwinkel *et al.*, 2010). As we have demonstrated that activation of the IFN-induction cascade by DIs can occur in the absence of protein synthesis and genome replication, the generation of progeny genomes is not required to activate the IFN response by DI-rich virus stocks. However, as our data and the data of others indicate that DIs are the predominant inducer of IFN during paramyxovirus infections (Baum *et al.*, 2010; Chen *et al.*, 2010; Killip *et al.*, 2011; Strahle *et al.*, 2006), for virus preparations that are DI-poor, the generation of progeny DI genomes during the replication of ND virus would be required to activate the IFN-induction cascade. The fact that virus protein synthesis was required for maximal levels of IRF3 activation by our working paramyxovirus stocks supports the idea that the replication of DI-poor stocks is required to generate and/or amplify the DIs that induce IFN. Our data therefore indicate that, for a given virus preparation, the requirement for protein synthesis and replication to activate the IFN-induction cascade is dictated by its DI content. Another interesting observation was that even virus preparations that are passaged by low multiplicity, so as to minimize the generation of DIs, still activate IRF3 at low levels in the presence of CHX, which could be due either to the replication of ND viruses activating IRF3, or to small amounts of DIs being present in these virus stocks. As all of our data indicate that ND viruses do not activate the IFN-induction cascade and that DIs are predominantly responsible for inducing IFN (Chen *et al.*, 2010; Killip *et al.*, 2011), and that our vM0 stock of PIV5-VΔC (which induced a small amount of IRF3 in the presence of CHX) contains low amounts of copyback DI genome (Killip *et al.*, 2011), we believe that the latter of these explanations is true. As DIs are generated rapidly during virus replication, it may be virtually impossible to generate virus stocks that are completely free from DIs, as we have observed small amounts of IFN induction even using viruses that have been plaque-purified (data not shown).

Whilst our experiments indicate that virus binding to cells is clearly insufficient to induce IFN, the integrity of the DI genomic RNA appears to be required for efficient IFN induction, as the IFN-inducing capacity of PIV5-VΔC DIs is sensitive to high doses of UV treatment. Whilst we are currently characterizing the DI-derived PAMPs in our PIV5-VΔC vM2 preparation, from the data presented here it is unclear whether it is the DI genomes themselves, RNA products made from these DI genomes, the exposure of DI virus genome to RIG-I during RNA synthesis or dsRNA formed by base-pairing of the RNA products with the DI genome template that is responsible for activating the IFN-induction cascade in the absence of virus protein synthesis. In this regard, it is interesting to note that a study using UV-inactivated NDV that had lost all of its infectivity showed that it retained its ability to synthesize RNA and induce IFN; further UV treatment of this virus reduced both RNA synthesis and IFN-inducing capacity at the same rate (Clavell & Bratt, 1971).

## Methods

### 

#### Cell and inhibitors.

A549, Vero, MRC-5, HSF (human skin fibroblast) and MG-63 cells (all from the European Collection of Cell Cultures) and their derivatives were grown as monolayers in Dulbecco’s modified Eagle’s medium (DMEM) supplemented with 10 % FBS at 37 °C. The generation and characterization of the A549/pr(IFN-β).GFP cell line were described previously (Chen *et al.*, 2010; Killip *et al.*, 2011). Cells were treated with CHX (used at 50 µg ml^−1^), actinomycin D (used at 5 µg ml^−1^) and MG132 (used at 10 µM) as indicated.

#### Viruses.

PIV5 wt (w3), PIV5-VΔC (He *et al.*, 2002) and MuV (Enders) were grown and titrated under appropriate conditions in Vero cells. SeV Z strain was grown and titrated in Vero cells in the presence of 2.5 µg *N*-acetyl trypsin (NAT) ml^−1^. The SeV Cantell preparation has been described previously (Johnston, 1981). DI-rich virus stocks, with the exception of MuV, are denoted vM followed by the number of sequential high-multiplicity passages required to generate them. DI-rich stocks of PIV5-VΔC (vM2) were generated as described previously (Killip *et al.*, 2011), and DI-rich preparations of SeV (vM5) were generated by sequential high-multiplicity passage in Vero cells in the presence of NAT. The MuV cl.3 preparation was plaque-purified from a DI-rich preparation of MuV (denoted MuV bulk) (Chen *et al.*, 2010; Young *et al.*, 2009). PIV5 and MuV infections were carried out in DMEM supplemented with 2 % FBS, whereas SeV infections were carried out in serum-free DMEM. For prediction of the fraction of cells in a population that are productively infected at different multiplicities, the Poisson distribution was used as follows: *P*(*k*) = e^−^*^m^m^k^*/*k*!, where *P*(*k*) is the probability that any cell is infected with *k* particles, *m* is m.o.i. (p.f.u. per cell) and *k* is the number of particles in a given cell (Condit, 2001). This formula simplifies to *P*(0) = e^−^*^m^* when estimating the number of uninfected cells in a population (i.e. when *k* = 0).

#### Immunoblotting, immunofluorescence and FACS.

Procedures for SDS-PAGE, immunoblotting, immunofluorescence and FACS have been described previously (Andrejeva *et al.*, 2004; Killip *et al.*, 2011). mAbs used included those raised against PIV5 NP (Randall *et al.*, 1987), PIV5 P (cross-reacts with MuV P) (Randall *et al.*, 1987), SeV NP, SeV P (a kind gift from Allen Portner, St Jude Children’s Research Hospital, Memphis, TN, USA), phospho-IRF3 (Ser396; Cell Signaling Technology) and actin (Sigma). Polyclonal antibodies used included those raised against ISG56, MxA and total IRF3 (all from Santa Cruz Biotechnology). Immunofluorescence was examined using either a Zeiss LSM 5 Exciter confocal microscope or a Nikon Microphot-FXA immunofluorescence microscope.

#### Analysis of IFN-β gene expression.

RNA from infected cells was prepared from 9 cm dishes of confluent cultures of MG-63 cells using the acid phenol method and analysed by RNase protection using probes for IFN-β and γ-actin (Enoch *et al.*, 1986).
